# Feline inflammatory aural polyps: a retrospective imaging-based evaluation

**DOI:** 10.3389/fvets.2026.1777956

**Published:** 2026-02-25

**Authors:** Alper Demirutku, Eylem Bektaş Bilgiç

**Affiliations:** Department of Surgery, Faculty of Veterinary Medicine, Istanbul University–Cerrahpaşa, Istanbul, Türkiye

**Keywords:** computed tomography, feline inflammatory aural polyps, magnetic resonance imaging, middle ear disease, traction avulsion, ventral bulla ostectomy

## Abstract

**Introduction:**

Feline inflammatory aural polyps (FIAP) are common inflammatory lesions of the middle ear in cats and may cause otic, vestibular, and neurological clinical signs. This retrospective study aimed to evaluate signalment, clinical presentation, lesion localization, diagnostic imaging findings, and surgical management in cats diagnosed with FIAP.

**Methods:**

Medical records of 154 cats diagnosed between 2019 and 2024 at a university veterinary hospital were retrospectively reviewed. Data regarding age, sex, breed, clinical signs, diagnostic imaging modalities, lesion characteristics, and surgical techniques were analyzed. Diagnostic evaluation included video otoscopy, computed tomography (CT), and magnetic resonance imaging (MRI).

**Results:**

Most affected cats were adults aged 2–7 years, and no sex predisposition was identified. Bilateral involvement was observed in 51.1% of cases, while 48.8% were unilateral, with no statistically significant association between lesion laterality and age or breed. In 98.45% of evaluated cats, inflammatory polyps involved both the dorsolateral and ventromedial compartments of the middle ear. Ventral bulla ostectomy (VBO), performed alone or in combination with traction avulsion, was the most frequently applied surgical technique. No recurrence was observed in cats treated with VBO during the follow-up period.

**Discussion:**

FIAP may present as unilateral or bilateral disease and frequently exhibit multicompartmental involvement. Multimodal imaging plays a crucial role in accurate diagnosis and surgical planning. Imaging-guided, individualized surgical management, particularly VBO when indicated, appears to provide favorable clinical outcomes.

## 1 Introduction

Feline inflammatory aural polyps (FIAP) are benign inflammatory proliferations that originate from the mucosa of the middle ear, nasopharynx, or external ear canal in cats (1, 2). They represent one of the most common causes of middle ear disease in feline patients and may result in a wide range of otic, vestibular, and neurological clinical signs (3–5). Although FIAP have traditionally been described as a disease of young cats, several studies have demonstrated that affected animals may range from juvenile to geriatric age groups (2, 6, 7).

The etiopathogenesis of FIAP remains incompletely understood. Chronic inflammation of the upper respiratory tract and middle ear has been proposed as a contributing factor, with extension of inflammatory processes through the auditory tube (5, 8). In addition, infectious agents have been identified in a proportion of cases, suggesting that secondary bacterial involvement may play a role in disease persistence and progression (7).

Clinical presentation varies depending on the anatomical extent of the lesion. Polyps involving the external ear canal or tympanic bulla are commonly associated with chronic otitis externa or otitis media, often accompanied by vestibular or neurological signs such as head tilt, nystagmus, ataxia, facial nerve paralysis, or Horner's syndrome (5, 7, 9). In contrast, polyps extending into the nasopharynx may lead to upper respiratory tract signs including nasal discharge, stertor, stridor, dysphagia, or respiratory distress (6, 9). Lesions confined to the middle ear may remain clinically silent for prolonged periods and are frequently diagnosed only after the onset of neurological manifestations (5).

Diagnostic imaging plays a pivotal role in the evaluation of FIAP. Conventional radiography has limited sensitivity for detecting middle ear disease and may fail to identify early or bilateral involvement (10). Advanced imaging modalities, particularly computed tomography (CT) and magnetic resonance imaging (MRI), allow detailed assessment of the tympanic bulla, middle ear compartments, and adjacent neurovascular structures, thereby facilitating accurate diagnosis and surgical planning (3, 11, 12). The increasing use of advanced imaging has also improved recognition of bilateral and subclinical disease, which may otherwise remain undetected (11, 13).

Several surgical techniques have been described for the treatment of FIAP, including traction avulsion, ventral bulla ostectomy (VBO), and less commonly lateral approaches or total ear canal ablation combined with bulla ostectomy (13–15). Traction avulsion alone has been associated with higher recurrence rates, particularly in cases with middle ear involvement (13). Ventral bulla ostectomy is widely regarded as the most definitive treatment option, providing improved access to the middle ear and lower recurrence rates when compared with conservative approaches (13, 16).

The aim of the present retrospective study was to evaluate signalment, clinical presentation, lesion localization, diagnostic imaging findings, and surgical management in a large cohort of cats diagnosed with FIAP. Additionally, this study sought to emphasize the importance of advanced imaging in identifying bilateral and multicompartmental disease and to support imaging-guided, individualized surgical decision-making.

## 2 Materials and methods

### 2.1 Study design

This retrospective study was conducted using medical records of cats diagnosed with feline FIAP between January 2019 and December 2024 at the Ear, Nose and Throat (ENT) Clinic, Department of Surgery, Istanbul University–Cerrahpaşa Faculty of Veterinary Medicine.

### 2.2 Case selection

Medical records were screened to identify cats with a definitive diagnosis of FIAP based on clinical examination findings, diagnostic imaging results, and intraoperative observations. Cats were included in the study if complete medical records were available, including signalment, presenting clinical signs, imaging findings, surgical approach, and follow-up information. Cases with incomplete data or uncertain diagnosis were excluded.

### 2.3 Clinical evaluation

Data collected from medical records included age, sex, breed, and presenting complaints. Clinical signs were categorized as otic, vestibular/neurological, or upper respiratory based on primary manifestation at the time of admission.

### 2.4 Diagnostic imaging

All cats underwent diagnostic evaluation including otoscopy or video otoscopy and advanced imaging. CT and/or MRI were performed to assess lesion localization, extent, compartmental involvement of the middle ear, and laterality. Video otoscopy was performed using HOPKINS^®^ telescopes (Karl Storz, Germany), while computed tomography was performed with a 128-slice multidetector CT scanner (Siemens SOMATOM Perspective, Siemens Healthineers, Germany) and magnetic resonance imaging was conducted using a 1.5-Tesla system (Siemens Magnetom FreeStar, Siemens Healthineers, Germany). Imaging findings were reviewed to determine involvement of the dorsolateral and ventromedial compartments of the tympanic bulla and to identify unilateral or bilateral disease.

### 2.5 Surgical management

Surgical techniques included traction avulsion, VBO, or a combination of both procedures. The choice of surgical approach was based on imaging findings, lesion localization, and surgeon preference. Intraoperative findings, completeness of polyp removal, and postoperative complications were recorded for each case.

The material removed from the middle ear, which filled the tympanic cavity, was submitted to the Laboratory of the Department of Pathology, Faculty of Veterinary Medicine, Istanbul University–Cerrahpaşa, for histopathological examination, and the results were recorded.

### 2.6 Statistical analysis

Descriptive statistical analysis was performed using standard statistical software. Continuous variables were summarized as mean and range, while categorical variables were expressed as frequencies and percentages. Associations between lesion laterality and age, sex, and breed were evaluated. Statistical significance was set at *p* < 0.05.

## 3 Results

Signalment characteristics were evaluated with respect to age, sex, and breed. Regarding age distribution, 82 cats (53.25%) were in the 2–7-year age group, 32 cats (20.78%) were between 6 months and 1 year of age, 22 cats (14.24%) were between 0 and 6 months of age, and 18 cats (11.69%) were 8 years of age or older. The study population consisted of 78 male cats (50.65%) and 76 female cats (49.35%). Breed evaluation revealed that 139 cats (90.26%) were mixed-breed, while 11 cats (9.74%) were purebred. Purebred cats included Scottish Shorthair (*n* = 3), British Shorthair (*n* = 1), Chinchilla (*n* = 1), Maine Coon (*n* = 1), Persian (*n* = 1), Ragdoll (*n* = 1), Bengal (*n* = 1), and Siamese (*n* = 2) cats (Table 1).

**Table 1:** Signalment characteristics of cats diagnosed with feline inflammatory aural polyps (*n* = 154).

**Characteristic**	**Group**	***n***	**%**
Age	0–6 months	22	14.24
	6 months−1 year	32	20.78
	2–7 years	82	53.25
	≥8 years	18	11.69
Sex	Female	76	49.35
	Male	78	50.65
Breed	Mixed breed	139	90.26
	Purebred	11	9.74

**Figure 1:**
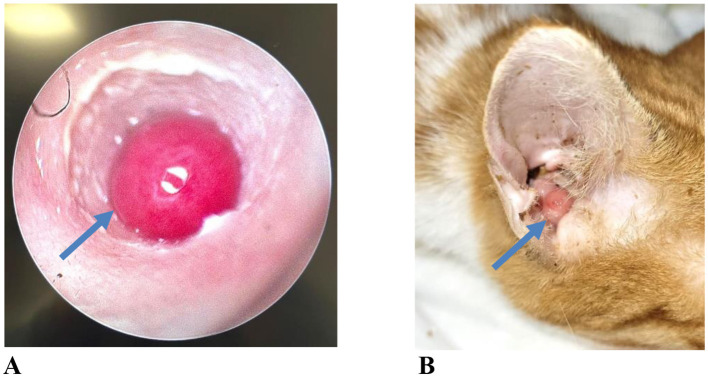
**(A)** Video otoscopic image of an inflammatory aural polyp extending into the external ear canal (case no. 121). **(B)** Gross appearance of an inflammatory aural polyp protruding from the right external ear canal, readily detectable on general physical examination without otoscopic evaluation (case no. 150).

**Figure 2:**
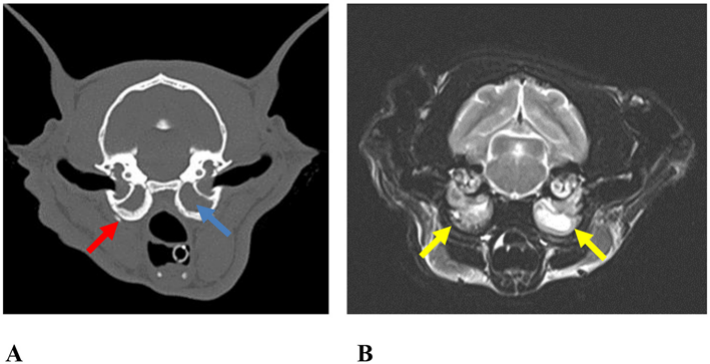
Axial images of the tympanic bulla from case no. 41. **(A)** Non-contrast computed tomography (CT) image showing soft tissue opacity occupying the tympanic bullae (blue arrow). Osseous wall thickening (red arrow) and increased bulla volume were observed bilaterally in the tympanic bullae. **(B)** T2-weighted magnetic resonance imaging (MRI) demonstrating a hyperintense signal within the same region (yellow arrows).

Multiple diagnostic modalities were used during the diagnostic process. Video otoscopy (Figure 1) was performed in 63 cats (40.91%), CT in 126 cats (81.82%), MRI in 51 cats (32.90%), and a combination of CT and MRI (Figure 2) in 49 cats (32.04%).

**Figure 3:**
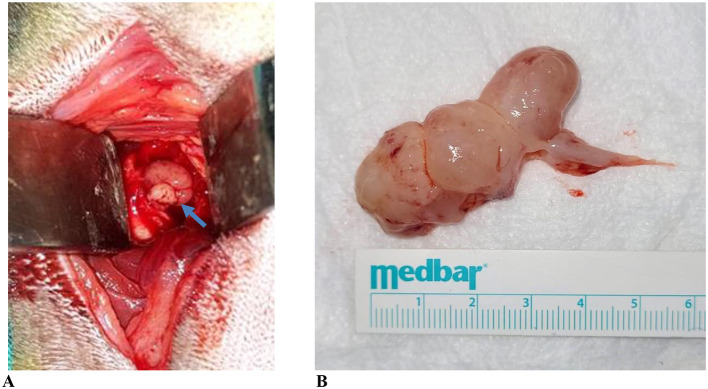
VBO and TA images of case no. 39. **(A)** Intraoperative view of an inflammatory aural polyp located in the ventromedial compartment following bulla osteotomy during VBO surgery. **(B)** Inflammatory aural polyp removed from the nasopharynx by TA. The polyp was observed to conform to the shape of the nasopharynx and to extend from the Eustachian tube with a distinct pedunculated appearance. The blue arrow indicates the inflammatory aural polyp located within the ventromedial compartment of the tympanic bulla during ventral bulla osteotomy.

**Figure 4:**
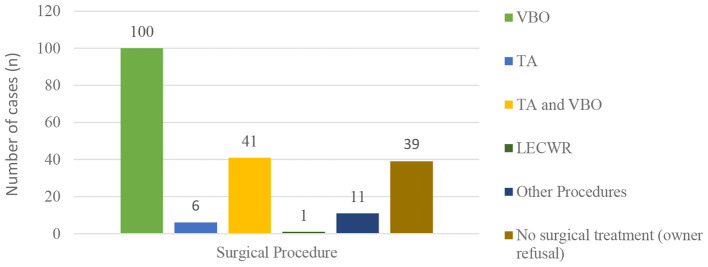
Frequency distribution of surgical procedures performed in cats with feline inflammatory aural polyps.

Various surgical approaches were applied for disease management. VBO was performed in 100 cats (64.94%), traction avulsion (TA) in 6 cats (3.90%), and combined TA and VBO (Figure 3) in 41 cats (26.62%). Lateral ear canal wall resection was performed in 1 cat (0.6%). Surgical treatment was not performed in 39 cats (25.32%) due to owner refusal. In the “other” treatment group, 11 cats underwent procedures outside standard surgical protocols for feline inflammatory aural polyps, including dorsal rhinotomy, nasal curettage, and nasal aspiration. In addition, medical treatment alone was administered in 2 cats (Figure 4).

With the exception of two surgically treated cases (Case Nos. 49 and 50), histopathological evaluation was performed on the material removed from the middle ear. According to the excisional biopsy results, all histopathologically examined cases were consistent with FIAP.

**Figure 5:**
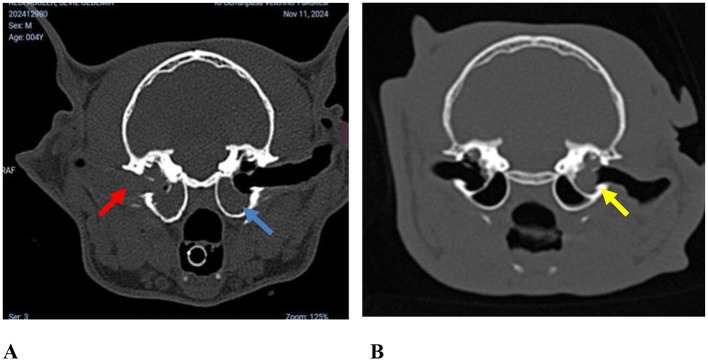
Axial non-contrast computed tomography (CT) images of the tympanic bullae. **(A)** In case No. 28, soft tissue attenuation filling both compartments of the left tympanic bulla with extension into the external ear canal is observed (red arrow), whereas the right tympanic bulla shows soft tissue attenuation limited to the ventromedial compartment (blue arrow). **(B)** In case No. 59, soft tissue attenuation is present only in the dorsolateral compartment of the tympanic bulla (yellow arrow) on axial non-contrast CT images.

**Figure 6:**
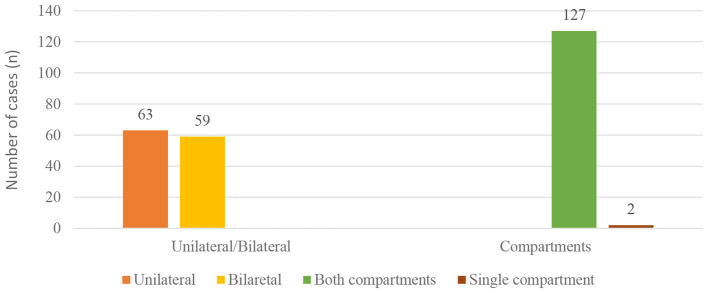
Lesion localization and compartment involvement in cats with feline inflammatory aural polyps.

Lesion localization was evaluated in 129 cats. Of these, 66 cats (51.16%) exhibited unilateral involvement, while 63 cats (48.84%) had bilateral lesions. Considering the bipartite anatomy of the feline middle ear, lesions involved both compartments in 127 cats (98.45%), whereas single-compartment involvement was observed in only 2 cats (1.55%). In case no. 28, the lesion was confined to the ventromedial compartment, while in case no. 59 (Figure 5), only the dorsolateral compartment was affected (Figure 6).

**Figure 7:**
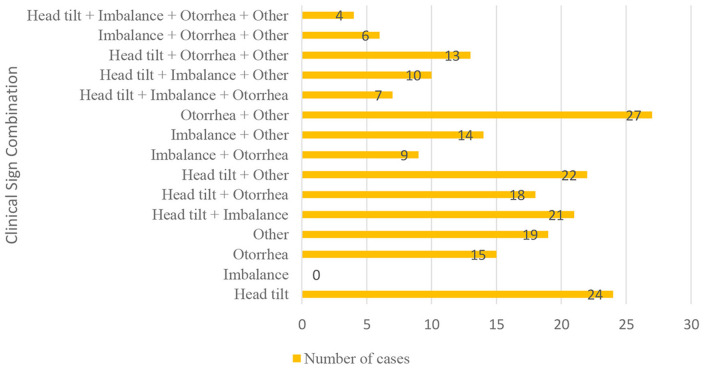
Summarizes single and combined clinical signs observed in cats diagnosed with feline inflammatory aural polyps.

Clinical signs were categorized as head tilt, imbalance, otorrhea, or other. The “other” category included Horner's syndrome (*n* = 2; case nos. 139 and 149), uncontrolled head movements, nystagmus, circling behavior, and upper respiratory tract–related signs, including nasal discharge (rhinorrhea), stertorous breathing, and sneezing. Head tilt alone was observed in 24 cases, otorrhea alone in 15 cases, and other signs alone in 19 cases; no cats presented with imbalance as the sole clinical sign. Concurrent clinical signs were frequently observed. Dual combinations included head tilt and imbalance (*n* = 21), head tilt and otorrhea (*n* = 18), head tilt and other signs (*n* = 22), imbalance and otorrhea (*n* = 9), imbalance and other signs (*n* = 14), and otorrhea and other signs (*n* = 27). Triple combinations consisted of head tilt, imbalance, and otorrhea (*n* = 7); head tilt, imbalance, and other signs (*n* = 10); head tilt, otorrhea, and other signs (*n* = 13); and imbalance, otorrhea, and other signs (*n* = 6). All clinical signs were observed simultaneously in 4 cases (Figure 7).

Chi-square (χ^2^) analysis was performed to evaluate the association between lesion distribution and age, breed, sex. No statistically significant difference was found in lesion laterality (unilateral vs. bilateral) among age groups (χ^2^ = 1.193; *p* = 0.755). Similarly, no statistically significant association was identified between breed and lesion laterality (χ^2^ = 0.136; *p* = 0.081). No significant association was found between lesion laterality and sex (χ^2^ = 0.272, *p* = 0.602; Table 2).

**Table 2:** Association between lesion laterality and age, breed, sex in cats with feline inflammatory aural polyps.

**Variable**	**Category**	**Unilateral (*n*)**	**Unilateral (%)**	**Bilateral (*n*)**	**Bilateral (%)**	***χ^2^***	***p***
Age	0–6 months	6	42.86	8	57.14	1.193	0.755
	6 months−1 year	14	51.85	13	48.15		
	2–7 years	35	55.56	28	44.44		
	≥8 years	8	44.44	10	55.56		
Breed	Mixed breed	35	46.05	41	53.95	0.136	0.081
	Other	28	60.87	18	39.13		
Sex	Female	30	47.62	33	52.38	0.272	0.602
	Male	36	53.73	31	46.27		

*P* < 0.05; Chi-square (χ^2^) analysis.

## 4 Discussion

FIAP have been reported to occur predominantly in young cats under 2 years of age; however, they may be encountered at any age, ranging from a few weeks to 15 years (2, 5, 9). In the present study, age was evaluated in four distinct groups, and FIAP were most frequently observed in cats aged 2–7 years, accounting for 53.25% of cases. Contrary to previously reported findings, the disease was more prevalent in mature adult cats in this cohort. Possible explanations for this discrepancy include the prolonged subclinical progression of the disease and the high population of stray cats in Türkiye. Considering that the majority of cases in the present study consisted of stray animals, the lack of routine clinical follow-up despite disease progression and presentation to veterinary care only after the development of pronounced neurological signs may have contributed to the higher prevalence observed in mature adult cats.

Previous studies have reported no specific sex predisposition for FIAP (2, 4, 13). Similarly, in the present study, the numbers of female and male cats were nearly equal, and sex was not found to have a significant effect on disease occurrence. Although these findings are consistent with existing literature, further studies with larger sample sizes are required to more definitively evaluate the relationship between sex and FIAP development.

The prevalence of FIAP in cats has been reported to range between 7% and 12%, with a possible genetic predisposition suggested for Siamese and Himalayan breeds (5, 14). In the present study, FIAP were identified in only two Siamese cats (case nos. 41 and 152), representing just 1.3% of the study population. This finding is not fully consistent with previous reports of breed predisposition. This discrepancy may be attributed to the predominance of mixed-breed cats in the study population, most of which were stray animals. Additionally, this observation supports the hypothesis that infectious and environmental factors may play a more substantial role in disease etiology than breed alone. Consequently, breed predisposition should not be considered a sole determining factor, and environmental conditions and upper respiratory tract infections may collectively contribute to FIAP development.

The diagnosis of otitis media (OM) and FIAP is based on the detection of fluid or mass lesions within the middle ear, typically utilizing otoscopic examination and advanced imaging modalities (15–17). Otoscopy is regarded as a fundamental diagnostic tool for the evaluation of ear canal, tympanic membrane, and middle ear pathologies (18). In the present study, otoscopy was routinely performed as part of the clinical examination; however, video otoscopy was preferred to allow visual documentation of lesions and facilitate owner understanding of disease severity. Video otoscopy was considered particularly valuable for identifying and visualizing polyps extending into the external ear canal and for improving owner compliance with recommended treatment.

CT and MRI are considered gold standard techniques for the diagnosis of OM and FIAP and are effective in identifying subclinical middle ear disease (15, 19). In the present study, video otoscopy, CT, and MRI were used for diagnostic purposes, and FIAP could not be definitively diagnosed using video otoscopy alone in any case. In selected cases, both CT and MRI were performed to improve diagnostic accuracy.

MRI has been reported to be particularly advantageous for identifying extra-auricular changes such as neoplastic formations and polyps (15). In this study, MRI was primarily utilized to evaluate the internal acoustic canal and meningeal structures in cases with prominent neurological signs. In contrast, CT remains indispensable for FIAP diagnosis and treatment planning due to its superior ability to depict bone and air densities at high resolution. These findings are consistent with previous studies emphasizing the diagnostic value of CT (19). Accordingly, the use of a multimodal imaging approach in FIAP and OM cases enhances diagnostic accuracy and enables more reliable surgical planning. CT provides detailed evaluation of osseous and aerated structures, while MRI allows comprehensive assessment of neurological tissues; therefore, their complementary use contributes significantly to thorough disease evaluation.

Several studies have reported that FIAP most commonly present as unilateral lesions (2, 7, 14). Nevertheless, bilateral involvement has also been described, with reported rates of approximately 24% (5) and 16% in studies with larger sample sizes (15). In the present study, bilateral involvement was observed in 48.84% of cases, resulting in nearly equivalent proportions of unilateral and bilateral disease. While this rate appears higher than previously reported, this finding should be interpreted with caution. One possible explanation may be the increased availability and more routine use of advanced imaging modalities, such as CT and MRI, which may facilitate the detection of subclinical or early-stage middle ear involvement. Consequently, middle ear disease may be identified before the development of overt neurological signs, including head tilt or imbalance. However, prospective studies with standardized imaging protocols and follow-up are needed to further clarify the true prevalence and clinical significance of bilateral involvement in FIAP.

The feline middle ear is anatomically divided into dorsolateral and ventromedial compartments by the tympanic septum. It has been reported that inflammatory infiltration in OM and FIAP initially remains confined to the dorsolateral compartment during acute stages and subsequently spreads throughout the tympanic cavity (16). However, there is no clear consensus regarding compartmental localization of FIAP. A prospective study reported that lesions frequently involved both compartments, although the limited number of cases restricted the generalizability of the findings (17). The results of the present study strongly support these observations, with 98.45% of cases exhibiting bicompartmental involvement. Single-compartment involvement was identified in only two cases, affecting the ventromedial compartment in one case (case no. 28) and the dorsolateral compartment in the other (case no. 59). These findings indicate that while FIAP may rarely remain confined to a single compartment, bicompartmental involvement represents the predominant pattern. The large sample size of the present study provides more robust and generalizable evidence regarding compartmental localization. Furthermore, this study is the first, to the authors' knowledge, to evaluate the association between lesion laterality (unilateral vs. bilateral) and age and breed, revealing no significant relationships. This finding suggests that factors other than age and breed may play a more critical role in FIAP etiopathogenesis.

Histopathological examination is considered the gold standard for the definitive diagnosis of feline inflammatory aural polyps and for differentiation from neoplastic or other chronic inflammatory middle ear lesions (13, 16). FIAP are macroscopically described as smooth-surfaced, pink-to-pale, pedunculated masses originating from the middle ear cavity and extending into the external ear canal, which can be observed during otoscopic or video-otoscopic examination (20). In the present study, a diagnosis of FIAP was established by histopathological examination in nearly all surgically treated cases. However, in cases in which surgical intervention was not accepted, the diagnosis was based on clear otoscopic visualization of the polyp, as described in the literature (20). Although histopathological examination is considered the gold standard for definitive diagnosis, the clinical examination and otoscopic findings obtained by an experienced clinician are also regarded as important components of the diagnostic process.

In addition, laterality assessment (unilateral vs. bilateral) could not be consistently standardized across all cases because advanced imaging (CT/MRI) was not available for every patient, and some cats were evaluated only by otoscopy. In several otoscopy-only cases, a polyp was clearly visualized on one side, whereas contralaterally there were tympanic membrane changes suggestive of middle ear involvement; however, the presence of a discrete polyp could not be confirmed. Therefore, these cases could not be confidently classified as bilateral. This highlights an inherent limitation of otoscopic assessment in determining true bilaterality and suggests that the prevalence of bilateral disease may have been underestimated in patients without cross-sectional imaging. Accordingly, the diagnostic value of CT and MRI for comprehensive evaluation of middle ear involvement and accurate laterality classification is further emphasized in the present study.

The clinical presentation of FIAP is highly variable. In addition to upper respiratory tract (URT) and otitis externa signs, vestibular manifestations such as head tilt, circling, nystagmus, and ataxia may be observed. Ipsilateral Horner's syndrome is also a commonly reported neurological finding in cats (20). FIAP-associated clinical signs often resemble those of otitis externa, including head shaking, ear scratching, and otorrhea (21–23). In the present study, otorrhea was the only otitis externa–related sign observed. In particular, FIAP was suspected in 15 cases presenting with purulent otorrhea, prompting advanced diagnostic imaging. Notably, no cases presented with head shaking or ear scratching as the primary complaint. While no cases presented with imbalance as an isolated sign, 24 cats were presented with head tilt alone. These findings suggest that FIAP-associated clinical signs in this cohort were more commonly related to otitis interna and that disease manifestations predominantly reflected middle and inner ear pathology. Additionally, Horner's syndrome was identified in two cases exhibiting only OM-related findings, indicating that disease may remain confined to the middle ear in some patients while still producing localized neurological signs. Overall, these results are consistent with previous studies demonstrating a wide clinical spectrum of FIAP and emphasizing the diagnostic relevance of neurological signs associated with otitis interna (20–23).

**Figure 8:**
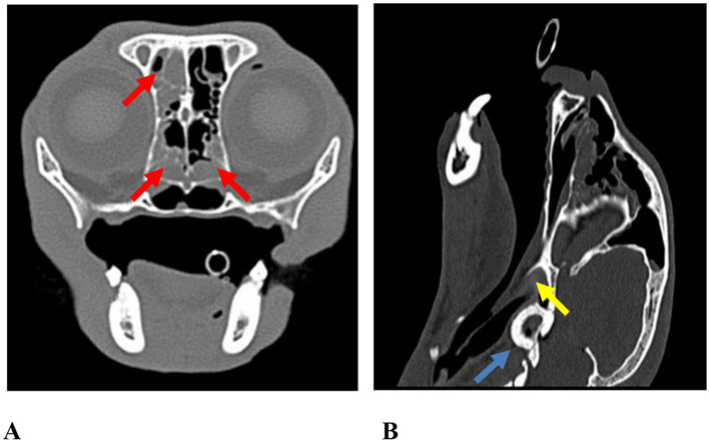
Autocontrast axial and sagittal CT images of case no. 149. **(A)** On the axial image, soft tissue density is observed within the left frontal sinus and bilaterally in the nasal cavity (red arrows). **(B)** On the sagittal image, soft tissue density is evident in the nasal cavity and tympanic bulla, accompanied by thickening of the bulla ossea (blue arrow) and an indistinct compression effect on the nasopharynx (yellow arrow).

Clinical signs of URT disease may also be observed in cats with FIAP, including nasal discharge, stertor, and sneezing (21, 23). In the present study, URT signs were classified within the “other” category, as they were less frequent and not always directly attributable to FIAP. Dorsal rhinotomy was performed in five cases (case nos. 27, 62, 128, 149, and 151), in which purulent rhinosinusitis was identified (Figure 8).

It has been suggested that mucosal swelling and recurrent sneezing associated with URT disease may increase nasopharyngeal pressure, facilitating Eustachian tube opening and subsequent disease development (21). Clinically, URT signs may predispose cats to secondary FIAP formation in chronic cases; however, the absence of FIAP in many URT infections supports a multifactorial pathogenesis. Accordingly, the findings of the present study indicate that FIAP development cannot be explained solely by this mechanism.

Chronic Eustachian tube dysfunction associated with inflammatory aural polyps may result in negative middle ear pressure and secondary inflammatory changes. Although CT and MRI findings were reviewed in this study, middle ear changes were not systematically evaluated using standardized criteria. This represents a limitation of the present study and may have influenced the comprehensive interpretation of imaging findings. Prospective studies with predefined imaging assessment protocols are warranted to better evaluate middle ear involvement in cats with inflammatory aural polyps.

Various surgical techniques have been described for FIAP treatment, including TA, VBO, myringotomy, lateral ear canal wall resection (LECWR), and combinations of total ear canal ablation and lateral bulla ostectomy (9, 13). In the present study, surgical procedures were categorized as VBO, TA, combined TA and VBO, LECWR, and other procedures. The “other” category primarily included dorsal rhinotomy, nasal curettage, and nasal aspiration performed for URT lesion management. TA alone was performed in cases with nasopharyngeal polyps, with subsequent VBO planned in separate sessions. In cases with external ear canal extension, TA and VBO were performed concurrently. Interventions targeting the nasal cavity in the “other” group were intended to prevent reinfection of the middle ear via the Eustachian tube. Myringotomy and total ear canal ablation were not performed in any case. The absence of myringotomy was attributed to bicompartmental involvement and large polyp volume, precluding complete removal. The availability of CT or MRI findings in all cases facilitated surgical decision-making and likely reduced the need for radical procedures such as total ear canal ablation.

Lateral approaches to the tympanic bulla are rarely applied in cats, with lateral bulla ostectomy primarily indicated for external ear neoplasia (23). In the present study, lateral approaches were not utilized, and polyps extending into the external ear canal were removed via TA. The primary reason for avoiding lateral approaches was the ability to differentiate polyps from neoplastic lesions through preoperative CT evaluation. The absence of tympanic bulla destruction and the independent extension of polyps along the external ear canal rendered lateral approaches unnecessary. Complications occurred in only one case (case no. 68), in which external ear canal perforation developed during TA, necessitating LECWR for correction. LECWR was not considered a primary treatment option for FIAP but was employed solely for complication management.

TA and VBO are the most commonly applied surgical techniques for FIAP treatment, with VBO associated with lower recurrence rates (0%−8%) (13). In the present study, TA and VBO were used either alone or in combination, depending on individual case characteristics. The findings suggest that both techniques serve as essential and complementary treatment modalities. Notably, no recurrence was observed in any case treated with VBO during the study period, supporting the effectiveness of this approach in reducing recurrence risk. In the present study, no recurrence was observed in cats treated with ventral bulla osteotomy during the available follow-up period. Postoperative follow-up was primarily limited to the early postoperative phase, with routine clinical re-evaluations performed for up to approximately 3 months. Beyond this period, further follow-up was based on representation of patients due to recurrent clinical signs. Therefore, the absence of observed recurrence should be interpreted in light of the existing follow-up conditions. This represents a limitation of the present study, as long-term and standardized follow-up data were not available for all cases. This limitation is particularly relevant given that a proportion of the study population consisted of stray cats, for which extended follow-up could not be consistently ensured. Prospective studies incorporating predefined follow-up intervals and long-term monitoring are warranted to more accurately determine recurrence rates following ventral bulla osteotomy in cats with inflammatory aural polyps.

## 5 Conclusions

In conclusion, this study provides clinically valuable insights into the diagnosis and management of feline inflammatory aural polyps based on a large case series, detailed advanced imaging evaluation, and systematic assessment of surgical approaches. In clinical practice, FIAP should be prioritized in the differential diagnosis of cats presenting with vestibular and neurological signs. The routine implementation of multimodal imaging techniques and individualized, imaging-guided surgical planning is essential for accurate diagnosis and optimal treatment. These findings contribute to the development of evidence-based clinical management strategies for FIAP and may serve as a solid reference for future prospective and multicenter studies.

## Data Availability

The data include patient and owner information; therefore, in accordance with the Turkish Personal Data Protection Law (KVKK), they cannot be shared with third parties. Requests to access the datasets should be directed to eylem.bilgic@iuc.edu.tr.
